# Psychoactive Drugs—From Chemical Structure to Oxidative Stress Related to Dopaminergic Neurotransmission. A Review

**DOI:** 10.3390/antiox10030381

**Published:** 2021-03-04

**Authors:** George Jîtcă, Bianca E. Ősz, Amelia Tero-Vescan, Camil E. Vari

**Affiliations:** 1 Department of Pharmacology and Clinical Pharmacy, Faculty of Pharmacy, George Emil Palade University of Medicine, Pharmacy, Science and Technology of Târgu Mureș, 540142 Târgu Mureș, Romania; george.jitca@umfst.ro (G.J.); camil.vari@umfst.ro (C.E.V.); 2 Department of Biochemistry, Faculty of Pharmacy, George Emil Palade University of Medicine, Pharmacy, Science and Technology of Târgu Mureș, 540142 Târgu Mureș, Romania; amelia.tero-vescan@umfst.ro

**Keywords:** oxidative stress, toxicity, bath salts, phenethylamine derivatives, dopamine

## Abstract

Nowadays, more and more young people want to experience illegal, psychoactive substances, without knowing the risks of exposure. Besides affecting social life, psychoactive substances also have an important effect on consumer health. We summarized and analyzed the published literature data with reference to the mechanism of free radical generation and the link between chemical structure and oxidative stress related to dopaminergic neurotransmission. This review presents data on the physicochemical properties, on the ability to cross the blood brain barrier, the chemical structure activity relationship (SAR), and possible mechanisms by which neuronal injuries occur due to oxidative stress as a result of drug abuse such as “bath salts”, amphetamines, or cocaine. The mechanisms of action of ingested compounds or their metabolites involve intermediate steps in which free radicals are generated. The brain is strongly affected by the consumption of such substances, facilitating the induction of neurodegenerative diseases. It can be concluded that neurotoxicity is associated with drug abuse. Dependence and oxidative stress are linked to inhibition of neurogenesis and the onset of neuronal death. Understanding the pathological mechanisms following oxidative attack can be a starting point in the development of new therapeutic targets.

## 1. Introduction

Amphetamine was first synthesized in 1887 by the chemist L. Edeleanu, being the leader of a group of compounds that have structural similarities and common biological effects. Amphetamine is currently approved by the FDA for the treatment of attention deficit disorder (ADHD) and narcolepsy, and some amphetamine derivatives are licensed for both ADHD and obesity treatment [1]. These substances are closely monitored by drug agencies because of their potential for abuse. The acute effects of the use of these substances include euphoria, increased alertness and psychomotor activity, decreased appetite, and even loss of appetite and hyperthermia. Long-term abuse causes psychotic, paranoid, aggressive and, above all, neurotoxic states [2]. Amphetamine derivatives promote oxidative stress by decreasing the gene expression of the antioxidant enzymes Cu-Zn superoxide dismutase (SOD), catalase (CAT) or glutathione peroxidase (GPx). Excessive heat production that induces hyperthermia and the pro-catabolic status facilitates the conversion of the enzyme xanthine dehydrogenase to the oxidizing form [3].

Synthetic derivatives of cathinones (β-keto-amphetamines), also called “bath salts”, are compounds with a stimulating effect. Their structure is derived from cathinone, a substance found in the khat plant, Catha edulis, a shrub cultivated in East Africa and South Arabia, consumed by local people for its mild stimulating effect [4]. Cathinones are used as cheap substitutes for other central stimulants, such as amphetamines or cocaine. Although the chemical structure of cathinones is similar to that observed in the structure of amphetamines, synthetic cathinones possess distinct psychopharmacological properties [5]. The structure of β-keto-amphetamine gives cathinones similar but not identical effects to amphetamine. Cathinones possess the ability to increase the concentration of catecholamines and serotonin (5-HT) by blocking specific transporters, but the psychopharmacological spectrum and illicit use differ [6]. If amphetamine is used more for its anorexigenic and stimulant properties, cathinones have evolved from “chemsex” to “slam” (by injection), due to entactogenic effects, to enhance sensory perceptions and sexual performance [6]. The mechanism of action of classical amphetamine derivatives and cathinones has been intensively studied. Currently, the proposed mechanisms for cathinones combine both cocaine-like properties (inhibition of dopamine (DA) and norepinephrine (NA) reuptake by blocking specific transporters) and amphetamine-like (interaction with the vesicular transporter of monoamines VMAT2, reversal of the reuptake direction and favoring efflux of neurotransmitters DA and/or NA).

Depending on the belongingness to one of the generation and the cellular transduction mechanism, cathinones can be grouped as follows [7,8,9,10,11,12]:

- cocaine-3,4-methylenedioxymethamphetamine (MDMA)—mixed group, non-selective monoamine reuptake inhibitors (cocaine-like), with preferential selectivity for dopamine transporter (DAT), but favoring the release of 5-HT like MDMA (e.g., mephedrone, methylone, ethylone, naphyrone);
- methamphetamine-like cathinones group, catecholamine reuptake inhibitors and DA liberators (e.g., methcathinone, flephedrone);
- pyrovalerone-derived compounds, catecholamine reuptake inhibitors, without a liberating action (e.g., 3,4-methylenedioxypyrovalerone also known as MDPV, 3,4-methylenedioxy-α-pyrrolidinobutiophene also known as MDPBP, α-pyrrolidinovalerophenone also known as α-PVP);

Being compounds intended for sale on the black market, “bath salts” are more often than not chemically pure compounds and do not represent a fixed combination of substances. Clinical data on the toxicity of pure cathinone consumption are rare, and case reports describe nonspecific symptoms, easily confused with other ones, and can mimic various conditions. Therefore, it is difficult to draw clear conclusions and appropriate therapeutic behavior. According to Drug of Abuse, A DEA Resource Guide, 2020 Edition [13], for drug addicts, the preferred routes of administration of “bath salts” are sniffing, but they can also be taken orally, smoked or injected. In the case of amphetamines, according to the same source, they are taken orally, injected or smoked. According to European Monitoring Centre for Drugs and Drug Addiction (EMCDDA 2020 report) [14], considering the route of administration of amphetamines, 52% reported sniffing, 15% reported oral consumption, 15% reported smoking, and 17% reported injection. Concerning the excitotoxicity of the two classes of substances, this is a picture made up of several successive events: massive release of glutamate (GLU), followed by the activation of specific receptors; as a result, there will be an increase in the intracellular calcium concentration. This signaling pathway will lead to activation of calcium-dependent enzymes, followed by the generation of reactive oxygen (ROS) and nitrogen species (RNS), and then to the activation of apoptotic mechanisms [2,15]. As a general mechanism of action, most synthetic cathinones have similarities to amphetamines—promoting DA, NA and 5-HT neuronal transmissions. Inhibition of catecholamine reuptake, similar to cocaine, is also described [16]. An attempt to establish cellular and molecular mechanisms generating ROS at the neuronal level and the involvement of amphetamines and cathinone derivatives in neurotoxicity may serve to discover therapeutic approaches to prevent the onset of negative consequences for human beings.

## 2. Materials and Methods

Data presented in this article were obtained following a literature search in four electronic databases: ScienceDirect, PubMed, PLOS, and Google Scholar. Inclusion criteria for articles were: title analysis, content of the abstract, and especially the information contained/included in the article. At the end of this review, data are presented on antioxidant therapy and how this therapy can have beneficial effects, reducing the toxic effects of these drugs diverted from medical use and/or trafficked illicit substances.

## 3. Results

This section presents data on chemical structure, the relationship between structure and pharmacological activity. At the same time, the influence of these psychoactive drugs on the levels of neurotransmitters is discussed, in particular DA, their mechanism of action being specified, correlated with the property of generating oxidative stress.

### 3.1. Transendothelial Blood Brain Barrier and Lipophilicity

In order to evaluate the neurotoxicity induced by cathinone and amphetamine derivatives, it is necessary to study the influences on the transporters and receptors of catecholamines; the result of the interaction is to modify the normal signaling pathways qualitatively and/or quantitatively. These biological targets are subject to cytotoxicity exerted as a result of the induction of oxidative stress. Thus, the neurotoxicity induced by these two classes of drugs is based on their ability to trigger inflammatory processes leading to irreparable degeneration (i.e., irreversible cytotoxicity). The relationship between neuronal toxicity and degeneration is closely related to the amount of drug reaching the central nervous system (CNS). The differences between the compounds (cathinone derivatives and non-β-keto-amphetamine analogues) are due to the different abilities to cross the blood-brain barrier (BBB). As the target of these drugs is the brain, the ability to reach the level of this organ depends not only on the route of administration, but also on their physicochemical properties (which determines the BBB penetration). In addition, it is possible that altered barrier function could cause the effects associated with neurotoxicity. It is known that molecules with a permeability index greater than 1 can easily cross the BBB. It is important to note that the compounds with a permeability index greater than or equal to 3 have a high permeability, and those with an index greater than 10 have a very high permeability. MDPV presents the highest degree of lipophilicity. This characteristic is due to the pyrrolidine ring and to the tertiary amino group which characterizes a compound with very low polarity and results in a high penetrating capacity of the BBB [17]. Abuse is associated with changes in the permeability of the BBB. In addition, excitotoxicity is added to this change which affects the distribution of the drug and is associated with a massive generation of ROS. This is the mechanism of induced neurotoxicity [18].

### 3.2. Influence of Neurotransmitter Transporters, Vesicular Transporters, and Receptors

Regarding the modification of the action of monoaminergic systems, this is one of the characteristics of amphetamine derivatives, and this influence will be discussed in more detail (including cathinone analogues).

Thus, the neurotoxicity exerted by amphetamine is due to the depletion of DA and 5-HT, inhibition of enzymes involved in the biosynthesis of neurotransmitters, tyrosine hydroxylase (TH) and tryptophan hydroxylase 2 (TPH-2), inactivation of DAT and serotonin transporter (SERT), and reduction of VMAT2 function [19], promoting apoptosis and neuronal degeneration.

Methamphetamine is known to have negative effects on the striatum (neurotoxicity due to dysregulation of mitochondrial function, neuronal energetic imbalance, overproduction of ROS, degeneration of axon terminals) especially on DA nerve endings. These effects could be a consequence of long term DA depletion and decreased DA synthesis due to reduced TH activity [2,17,20,21,22].

Although the number of studies is limited, the effects of β-keto-amphetamine compounds appear to be more complex, influencing more neurotransmissions; methcathinone is known to be selective in the release of mediators (it has the ability to release DA predominantly, but the serotonergic mechanism cannot be ruled out either) [9,17,23,24]. At repeated doses, the activity of DAT, SERT, enzymes involved in the biosynthesis of catecholamines, TH, and TPH-2 decreases. Regarding mephedrone, it has the property of releasing DA (similar to methamphetamine, but much weaker), but the influence on DA neurons is greater than that exerted by MDMA. Microdialysis studies performed on rat brains showed that methamphetamine and mephedrone increase the extracellular level of 5-HT (as opposed to amphetamine), which can be considered a qualitatively different pharmacological effect [8,25,26].

Experimental data conducted on brain synaptosomes of rats demonstrate that mephedrone and methylone are non-selective inhibitors of the DAT, norepinephrine transporter (NET) and SERT, causing an increase in the extracellular level of DA and 5-HT [7,8,11,25,27,28,29]. Microdialysis experiments show that pyrovalerone and MDPV derivatives increase the extracellular concentration of DA in the *nucleus accumbens*, (NAc) [25], these derivatives having a higher inhibitory capacity against DAT, being among the most important inhibitors [9,25,29]. On the other hand, these compounds do not have the property of stimulating the release of DA [7,17,30].

To sum up, DA is the main factor generating negative effects, having a stronger influence compared with 5-HT. This is due to the increased susceptibility of DA neurons to enzymatic and non-enzymatic oxidative processes [31,32,33,34].

### 3.3. Mechanisms of Action

Both groups of psychotropic drugs discussed, due to their psychostimulant effects, are classified as responsible for misuse and abuse. These effects can occur through two distinct mechanisms:

- cocaine-like mechanism (inhibition of DA and NA reuptake following DAT and NET blockade) [35,36];
- amphetamine-like mechanism (favoring the outflow of DA and/or NA neurotransmitters) [37,38];

In the case of amphetamine compounds, two other accessory mechanisms that contribute to the occurrence of neurotoxicity have been discovered:

- VMAT interaction (modifying the pH by decreasing the proton gradient along the membrane, inducing the release of monoamines from the vesicles to the cytosol) [39,40,41,42];
- inhibition of monoamine oxidase (MAO) and/or catechol-ortho-methyltransferase (COMT) [43,44,45,46];

Unlike amphetamine derivatives, mephedrone and methylone lack affinity for the VMAT transporter. This difference is reflected in a lower degree of neurotoxicity. Concomitant use of methamphetamine and mephedrone or methylone worsens the toxic effects (addition of side effects); nevertheless, the use of methamphetamine with MDPV has been shown to have protective effects, because, MDPV, by blocking DAT mediated transport (inward or outward), blocks methamphetamine-induced dopamine release [17,47].

Pyrovalerone derivatives such as MDPV (a potent DAT inhibitor), show their psychotic effects at lower doses. Also, they have a high degree of lipophilicity, crossing the BBB more easily. The increased potency of inhibiting DAT and SERT is related to the addictive potential, whereas lipophilia is linked to the ability to cross the BBB, which may be correlated with the latency of psychoactive effects. Also, the promotion of DA neurotransmission by the psychoactive drugs (e.g., MDPV, α-PVP, α-PPP) is due to inhibition of DAT and accumulation of DA in the synaptic cleft. Moreover, 5-HT accumulated in the synaptic cleft as a result of the interaction with SERT (e.g., mephedrone, naphyrone, MDMA) enhances DA activity through post synaptic 5-HT2A receptors, which favors the release of DA [7,8,9,11,48]. The 5-HT and DA interaction in NAc is complex, the addictive properties can be mediated by the increase of the DA level in the ventral striatum (the increase in DA release is directly responsible for activating the reward system) [49,50]. The vasoconstriction and hyperthermia induced by compounds such as amphetamine, methamphetamine, methcathinone, mephedrone or flephedrone is the consequence of the stimulation of peripheral α1A and α2A adrenergic receptors. Thus, we can explain the toxic sympathomimetic effects observed regularly after intoxication [9].

### 3.4. Structure Activity Relationship

#### 3.4.1. Cathinone Derivatives

**Figure 1:**
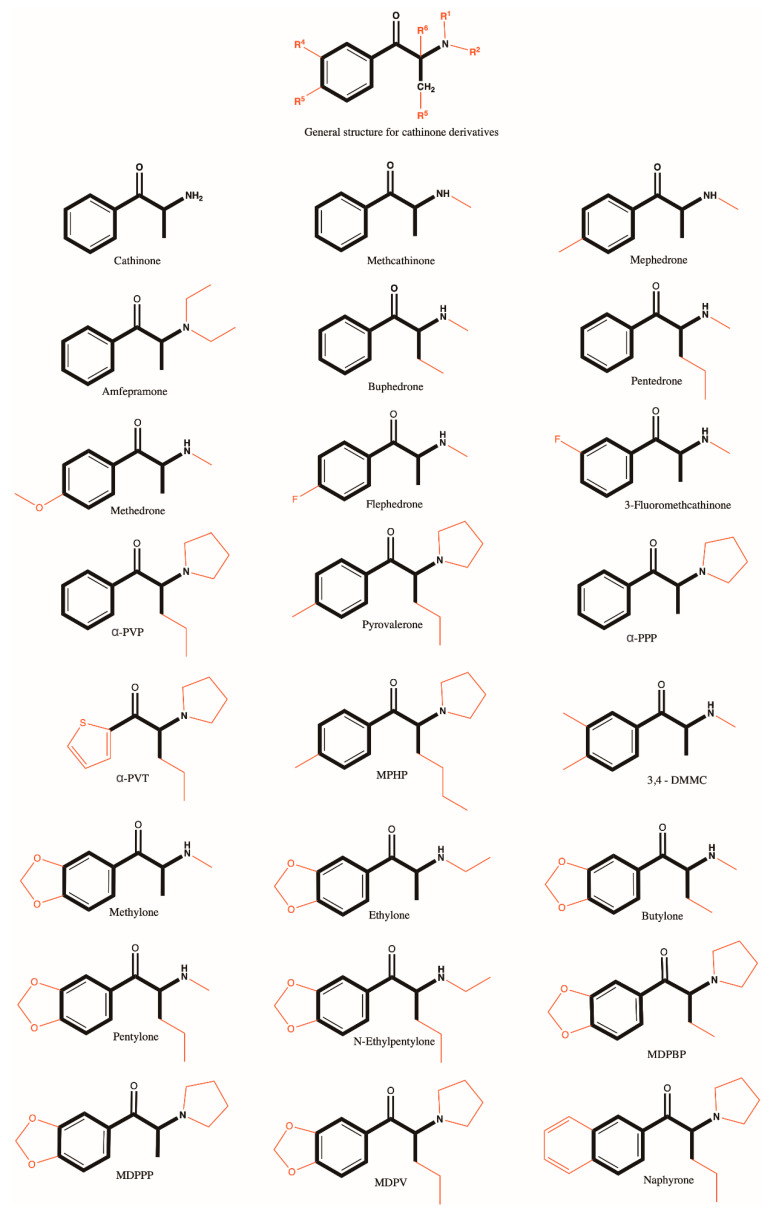
Chemical structure of cathinone derivatives.

The study of the relationship between structure and activity aims to establish the influence of chemical structure on biological action (see Figure 1 for chemical structures). The purpose is to identify the substituents which modify the activity (in particular the type of action, the potency, the affinity). For the compounds examined, the actions of a number of agents may be related to their ability to activate a specific receptor in the brain. The reason why it is important to know the structure-activity relationship (SAR) is that the number of new synthetic compounds derived from cathinone is constantly increasing. Thus, the report of EMCDDA 2020, identifies approximately 100 new derivatives of synthetic cathinones in 2018, compared with approximately 10 structures in 2008. In Europe, approximately 36% of the amount of confiscated new psychoactive drugs is dominated by syntethic cathinones (quantitatively, synthetic cathinones and cannabinoids accounting for more than three quarters of the total). Undoubtedly, new compounds will continue to appear due to the knowledge regarding the chemical structure pharmacological effect relationships [24].

In the case of substituted 4-MCAT derivatives (e.g., mephedrone, flephedrone), the selectivity for DAT vs SERT may explain the addictive behavior (dopamine-related reward system), relative to the hallucinogenic potential, which is not necessarily related to dependence.

Butylone, the compound with the ethyl group in the α position of the 2-methylamino-1-(3,4-methylenedioxyphenyl) propan-1-one (MDMC) side chain (instead of the methyl group) acts as an inhibitor of DA, NA, and 5-HT reuptake by blocking the three specific transporters DAT, NET, and SERT. The presence of the α-ethyl group (specific to MBDB and butylone structures) decreases the ability to induce euphoria and thus the risk of abuse due to a reduced ability to interact with the NET carrier. Thus, the α-methyl group (from the structure of MDMA or methylone) is responsible for the preferential inhibition of NET and the high potential for abuse [9].

In addition, the increase in chain length (change of methylenedioxy from MDMC to ethylendioxy, specific for EDMC) decreases the ability of substances to cause the release of the three mediators [51].

Naphyrone is obtained if the methylenedioxy ring of MDPV is replaced by an aromatic (phenyl) ring. This compound behaves as a blocker of the three carriers (DAT, NET, SERT); however, the blocking action occurs preferentially on SERT and to a lesser extent on NET and DAT (the main difference between naphyrone and MDPV) [9]. A pyrrolidine ring and a flexible alkyl chain in the α position (e.g., MDPV) can be added to block the DAT transporter. The introduction of the 3,4-methylenedioxy group does not have major consequences, as demonstrated by Marusich et al. [52]. As a result, the removal of the 3,4-methylenedioxy group from MDPV to form α-PVP has negligible effects on the ability to block DAT or NET in rat brain synaptosomes. Furthermore, the introduction of a pyrrolidine ring to any cathinonic analogue (e.g., MDPV, MDPBP, 3,4-methylenedioxy- α-pyrrolidinopropiophenone also known as MDPPP) can be used to confer the specific blocking properties of both catecholamine transporters (DAT, NET) [10,53,54,55,56].

Conclusions resulting from the study of SAR are difficult to draw, most of the studies having focused on the determination of the pharmacokinetic (administration route)/pharmacodynamic (pharmacological effects) properties of the synthesized compounds, without making a real initial selection based on potential properties through in silico modeling. While there are many studies on in silico modeling of SAR in the case of cathinones, they remain in the field of academic research. On the black market, the selection of new compounds is based on the already discovered old structures, the goal not necessarily being to change the properties (lipophilicity, selectivity for DAT, SERT, etc.), but above all to avoid the introduction of compounds on the prohibited list. As a result, most of the new compounds, later classified into generations, were designer molecules of the old compounds, the list having to be constantly updated by the legislator [57,58,59,60,61,62,63]. The DAT vs SERT ratios of synthetic cathinones are presented in Table 1.

#### 3.4.2. Amphetamine Derivatives

**Figure 2:**
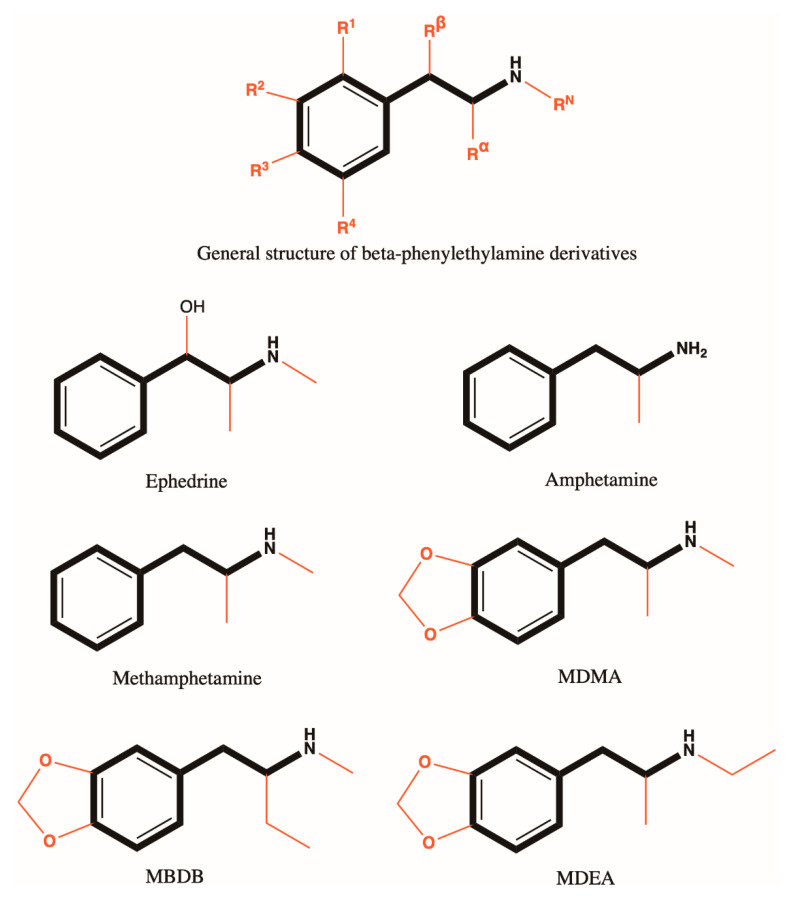
Chemical structures of amphetamine derivatives.

For amphetamine derivatives with general structure presented in Figure 2, the introduction of the ethyl group in the α position (e.g., MBDB) decreases the possibility of influencing the central dopaminergic pathways. Alkylation of the amino group (e.g., methamphetamine, MDMA, MBDB, 3,4-methylenedioxy-N-ethylamphetamine also known as MDEA) alters the intracerebral distribution of the compound, but also greatly influences the pharmacokinetic parameters [48,70]. Substitutions in the aromatic nucleus change the type of effect, with a diversification of the biological response: fenfluramine behaves like a sedative and produces dysphoria, while MDMA is a central stimulant drug which produces marked euphoria [56,71,72]. There are also compounds with an α, α’-dimethyl structure derived from MDMA or MBDB (such as 3,4-methylenedioxyphentermine). In addition, alkylation to the amino group (for example, the N-methyl analogue) leads to a special pharmacological profile—they do not produce the release of 5-HT from the synaptosomes of the rat brain [56]. Since the discovery of the class of amphetamine derivatives, it has been demonstrated that the substitution of the aromatic ring by a bulky group increases the selectivity for SERT to the detriment of DAT; thus, 3-norfenfluramine behaves as a preferred substrate for SERT as opposed to amphetamine, the parent compound [73,74]. The chemical structures of amphetamine derivatives are presented in Figure 2.

Based on the relationship between structure and activity, amphetamine analogues and cathinone derivatives have similar pharmacological/psychopharmacological effects. The similarities consist of the main structural core (β-phenylethylamine) which confers peripheral and central sympathomimetic action. The extension in the hydrocarbon chain increases the lipophilicity of the molecule and provides protection against enzymatic degradation (e.g., α-PVP, Pyrovalerone, Pentylone, N-ethylpentylone, MDPV, MBDB) [70].

The therapeutic target for drugs (amphetamine and cathinone derivatives) are represented by DAT, NET, and SERT. The cause of this similarity is the presence of a common pharmacophore in the molecule. Alkylation of the amino group (addition of an N-methyl group to amphetamine) alters the selectivity to transporters (increases to SERT vs DAT, does not change to NET)—also, the introduction of the methylenedioxy group (in the case of MDMA) leads to an increased selectivity for SERT (which explains the differences in psychotoxicity between amphetamine and MDMA) [75].

The β-ketone analogue of methamphetamine (methcathinone) has diminished adrenergic effects, maintaining the intensity of dopaminergic ones. For substances with high affinity for SERT, the way to induce oxidative stress is different, because 5-HT is not directly involved in the generation of free radicals [76,77]. Increased 5-HT levels can lead to the onset of serotonin syndrome (manifested as tachycardia, hypertension, hyperthermia) and it can have a significant social impact due to the fact that the accumulated 5-HT has the ability to stimulate postsynaptic 5-HT2A receptors leading to increase DA release in NAc [49,50]. These effects can be indirectly correlated with oxidative stress. The more recurrent need for higher doses due to more frequent misuse also increases the availability of DA, as the selectivity towards SERT is only preferential, nevertheless, the release and inhibition of DA reuptake still remain relatively noticeable [70]. The DAT vs SERT ratios of amphetamine derivatives and cocaine are presented in Table 2.

Activation of the central dopaminergic and serotonergic pathways by synthetic derivatives of cathinone and amphetamine derivatives may act as a trigger for acute psychotic reactions. These are common in consumers of “bath salts” who previously had a normal psychological profile. Given the illicit origin of most of the compounds, their synthesis takes place in clandestine laboratories. Consequently, the presence of impurities (e.g., manganese) [78,79,80,81,82,83], has been described and chronic exposure has additive, “impurity-related” toxicity. In addition, the “finished product” is often diluted with other psychotropic compounds (e.g., cannabinoids, ephedrine, caffeine, opioids, benzodiazepines, etc.). Consumption of such a mixture can lead to both acute poisoning and chronic exposure, both of which have a multifactorial etiology [84,85,86,87].

### 3.5. Biochemical Mechanisms: Dopamine, Oxidative Stress, and Cytotoxicity

Nowadays, the dependence and consumption of illegal drugs is a major social concern, not only in developing countries, because of medical complications in different organs (e.g., brain, heart, liver, and kidneys) [88]. This toxicity is studied from the perspective of a new approach, by evaluating the oxidative stress, manifested by macromolecular degradation, lipid peroxidation, functional incapacity and cell death by apoptosis [89].

The beneficial effects of a certain level of oxidative stress are undeniable given their involvement in the phagocytosis processes [90,91] and cell signaling [92]. At the same time, recent studies show that ROS play a key role in apoptosis [93], being part of the transduction signal during this process [94]. An abnormal production exceeds the endogenous antioxidant capacity (“damage control”).

For obvious reasons, the brain is one of the organs highly affected by oxidative stress and despite the fact that it represents only 2% of the total body weight, it consumes about 20% of the inspired oxygen and generates an increased amount of free radicals. Moreover, cerebrospinal fluid contains transition metals like iron or copper that catalyze reactions in which free radicals are generated [95]. The mechanisms through which free radicals damage neuronal tissue are not exactly known, but it is commonly accepted that the permeability of the BBB and the normal morphology of the brain are affected [96].

Neurotransmitters, such as GLU or DA, are also involved in the generation of free radicals. For example, the GLU action on NMDA receptors produces an influx of calcium ions that activates nitric oxide synthase (NOS), and nitric oxide (NO) is a precursor of peroxynitrite. During enzymatic and non-enzymatic oxidation of DA, free radicals are generated in DA neurons from various areas of the brain, the most important of which is the nigrostriatal pathway, which projects from the substantia nigra to striatum, involved in Parkinson’s disease and iatrogenic parkinsonism [97].

DA is an extremely important neurotransmitter in the CNS. It is released at the level of the reward system, especially in the NAc, being responsible for the well-being produced by euphoric substances. However, DA has other functions through dopaminergic pathways and neural connections through which this neurotransmitter reaches many areas of the CNS. The feeling of pleasure and reward induced by DA is controlled by mesolimbic dopaminergic pathways that start from the ventral tegmental area (VTA) located in the *midbrain*. From here, through the projections of DA neurons, the nerve impulse reaches the NAc, the place where DA produces these positive feelings, including the euphoria induced by psychoactive substances [98].

The mesocortical pathway also begins in the VTA, but, in this case, the projections of DA neurons reach the medial *prefrontal cortex* (mPFC) where they control cognition, decision-making and memory, especially working memory. This explains why certain psychoactive substances (e.g., amphetamines) increase, in the first phase, the capacity to work. Another dopaminergic pathway (the nigrostriatal pathway) controls involuntary movements and projects from the *substantia nigra* to *striatum* (*caudate nucleus* and *putamen*). However, DA also has endocrine effects through the tuberoinfundibular pathway from the *hypothalamus* (*arcuate* and *periventricular nuclei*). At this level, DA acts as a PIF (prolactin inhibitory factor) allowing negative control of the release of prolactin from the anterior pituitary gland [99].

For example, the concrete mechanism by which MPTP (1-methyl-4-phenyl-1,2,3,6-tetrahydropyridine) exerts its selective toxic action on DA neurons and causes iatrogenic parkinsonism is only partially understood. MPTP, being a compound with a high lipid/water partition coefficient, readily crosses the BBB. Benefiting from an amphiphilic structure, it is taken up by astrocytic lysosomes [100] and oxidized to MPP+ (1-methyl-4-phenylpyridinium) by MAO-B [101].

It is then preferentially captured by DA neurons via DAT. Here, MPP+ blocks the electron transport chain (ETC) by inhibiting complexes I and III. As a result, the amount of ATP decreases, the intracellular concentration of Ca^2+^ increases. ROS are generated in an autocatalytic manner and neuronal apoptosis is activated [102].

### 3.6. Cathinone Derivatives

**Figure 3:**
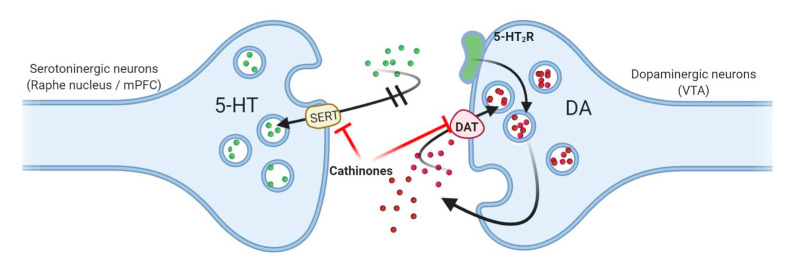
The general mechanisms proposed for promoting dopaminergic neurotransmission by cathinones. Cathinones inhibit the reuptake of serotonin (5-HT) and dopamine (DA) from the synaptic cleft by blocking the activity of transporters for 5-HT (SERT) and DA (DAT), and subsequently leading to accumulation of 5-HT which stimulates postsynaptic 5-HT_2A_ receptors (5-HT_2_R) favoring the release of DA in the synaptic cleft (and which then remains at this level and cannot be recaptured). Created with BioRender.com (accessed on 22 January 2021).

Because of the strict legal regime of amphetamine-type drugs and MDMA [103], drug abusers turned to other substances that are easier to obtain, known as “bath salts” [104] among which is mephedrone, a compound with β-keto-amphetamine structure. Similar to amphetamine analogues, this substance acts at the cortical and striatal level, or on the NAc [105] by modulating DA and 5-HT transmission [106,107,108,109,110,111], inhibiting reuptake [8] and stimulating the release of monoamines from vesicles [112,113], as shown in Figure 3.

Animal studies suggest that mephedrone stimulates motor functions and chronic use causes a progressive loss of 5-HT and DA neurons in the hippocampal and striatal area [111,112,113]. The cause of this massive damage of DA neurons has not been elucidated yet, but it has been hypothesized that oxidative stress caused by ROS and RNS could be a major contributor. The origin of these reactive species are the neurotransmitters, especially DA and the influence of psychoactive agents on the mitochondrial ETC [111,114,115,116,117,118,119]. In spite of antioxidant systems, free radicals accumulate causing immediate molecular damage (e.g., protein carbonylation, lipid peroxidation) [17] and degradation of cellular organs playing an important role in the development and progression of neurodegenerative diseases [111]. In adolescents, oxidative stress generated by abuse of psychoactive drugs affects both cortical neurons and transmission to subcortical structures, with consequences on cognitive function [120,121]. In general, the consumption of psychostimulant drugs exerts pro-oxidative effect and, in the *hippocampus* and *prefrontal cortex* [113], it decreases the total antioxidant capacity, the activity of antioxidant enzymes and it also increases the concentration of malondialdehyde (MDA) [122,123], as the brain contains considerable amounts of lipids and transition metals, resulting in alteration of interneuronal transmission [121,122]. Among the clinical manifestations, which appear after the consumption of substances with β-keto-amphetamine structure, are hypokinesia and dystonia, suggesting modifications of the extrapyramidal system, similar to Parkinson’s disease [124], with the remark that the use of mephedrone does not produce tremor-at-rest [79]. A possible cause of these symptoms is the accumulation of manganese [125] used in the synthesis of the drug [126], in the internal globus pallidus [127], and in the pars compacta of substantia nigra [128], where it exerts various cytotoxic effects including generation of free radicals and apoptosis in the corpus striatum [129,130]. There is no antidote for manganese or mephedrone intoxication and the classic antiparkinsonian medications are not effective in this syndrome [79,131].

### 3.7. Amphetamine Derivatives

**Figure 4:**
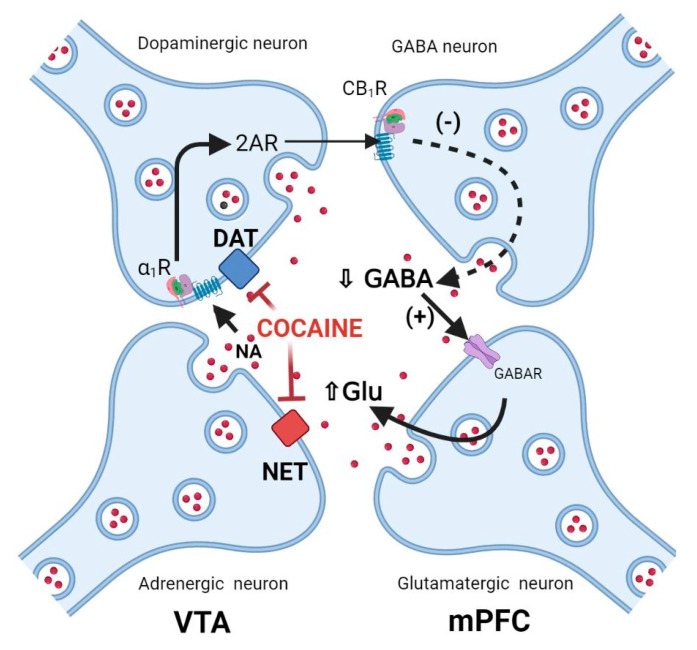
The proposed mechanism for promoting glutamatergic neurotransmission by cocaine—cocaine inhibits the reuptake of dopamine (DA) and norepinephrine (NA) due to the inhibition of specific dopamine transporter (DAT) and norepinephrine (NET) transporters in *medial prefrontal cortex* (mPFC). NA in the synaptic cleft stimulates α_1_ (α_1_R) receptors in DA neurons by promoting the release of endocannabinoids (2-arachidonoylglycerol, 2AR) which, after binding to CB_1_R receptors, inhibit gamma-aminobutyric acid (GABA) release in the synaptic cleft. A lower concentration of GABA promotes the release of glutamate (GLU) due to the suppression of the inhibitory effect of GABA on the release of GLU. Created with BioRender.com (accessed on 22 January 2021).

**Figure 5:**
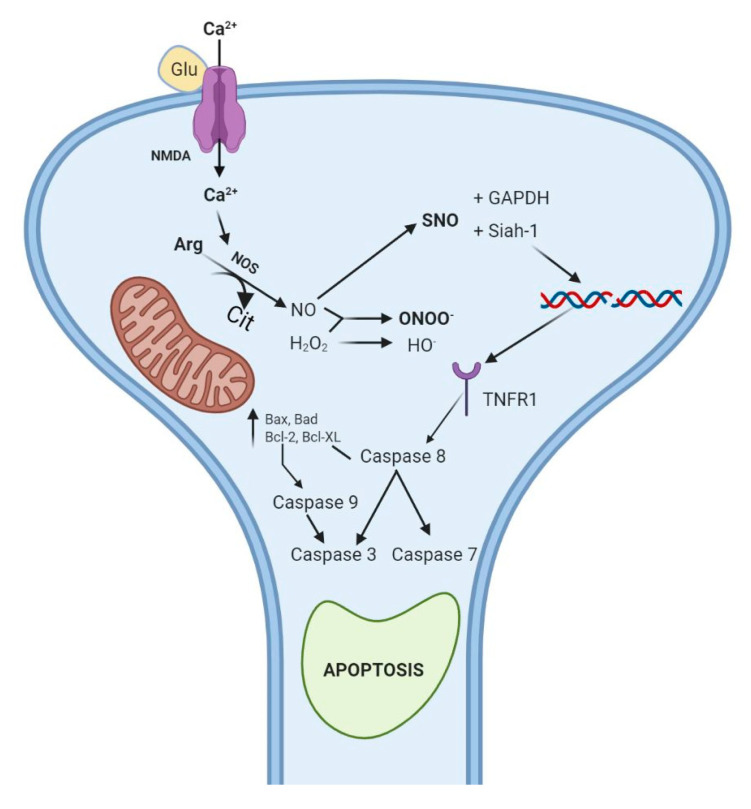
Glutamate (GLU) mediated neurotoxicity. GLU stimulates NMDA receptors by promoting the influx of intracellular Ca^2+^. Calcium increases nitric oxide-synthase (NOS) activity with increasing intracellular concentration of nitric oxide (NO). S-nitrosothiols (SNO) together with reactive oxygen species (ROS) resulting from the Haber Weiss/Fenton reaction, forms the ONOO^-^ radical which after binding to glyceraldehyde-3-phosphate dehydrogenase (GAPDH) interacts with Siah1 the complex formed by stimulating the transition/translation process with tumor necrosis factor (TNFR_1_) receptor overexpression at the cell surface. This favors the action of TNFα and the initiation of the apoptotic process. Created with BioRender.com (accessed on 22 January 2021).

In case of amphetamine and methamphetamine use, similar to mephedrone, the immediate effects are caused by interference with DA neuronal transmission [132]. These substances penetrate the neuron and cause a massive release of the neurotransmitter into the synaptic cleft. Studies in rodents show that amphetamines increase the levels of oxidative stress markers such as MDA, SOD, glutathione (GSH/GSSG), 2,3-dihydroxybenzoic acid in the *cortex, corpus striatum* [133], and *hippocampus* [134]. Moreover, toxic doses of methamphetamine inhibit the ETC, by interfering with all four complexes, in the *corpus striatum*, *hippocampus*, *amygdala, nucleus caudatus*, and *prefrontal cortex*, being incriminated in the development of neurodegenerative diseases [135,136,137]. Neuronal death in these areas occurs by apoptosis [138] as a consequence of the modified ratio between pro-apoptotic (Bax, Bad) and anti-apoptotic proteins (Bcl-2, Bcl-XL) resulting in the activation of caspases 9 and 3 [139,140]. Amphetamine and/or cocaine-induced neurotoxicity [141] is mediated by the glutamatergic system (Figure 4), by activation of NMDA receptors [139,142], following intracytoplasmic influx of Ca^2+^ [133,143,144] and activation of NOS. Following the increase in the concentration of NA in the synaptic cleft, there will be an increase in the concentration of GLU in mPFC neurons (the inhibition of the inhibitory control of neurotransmitter release by gamma-aminobutyric acid, GABA). This increase can be facilitated, for example, by inhibiting the reuptake of NA by cocaine. Consequently, NA activates α1 adrenergic receptors located in DA VTA neurons. This process promotes the release of 2-arachidonylglycerol (2-AR) into the synaptic cleft, thus activating the endogenous cannabinoid pathway. The lipid mediator acts on postsynaptic cannabinoid receptors (CB1R) preventing the release of GABA. Thus, the negative control is suspended and there will be an increased release of GLU [145]. Due to the activation of NOS following Ca^2+^ influx, the newly formed compound can generate free radicals, especially peroxynitrite [146], which interacts with the hydroxyl radical formed by the Haber-Weiss/Fenton reaction [137,147,148,149]. Besides the vasodilating effect following the activation of cGMP, NO can nitrosylate proteins that modulate the apoptotic process. One such protein is glyceraldehyde-3-phosphate dehydrogenase (GAPDH) which enters the nucleus in nitrosylated form and is able to interact with Siah1 [150] as shown in Figure 5.

**Figure 6:**
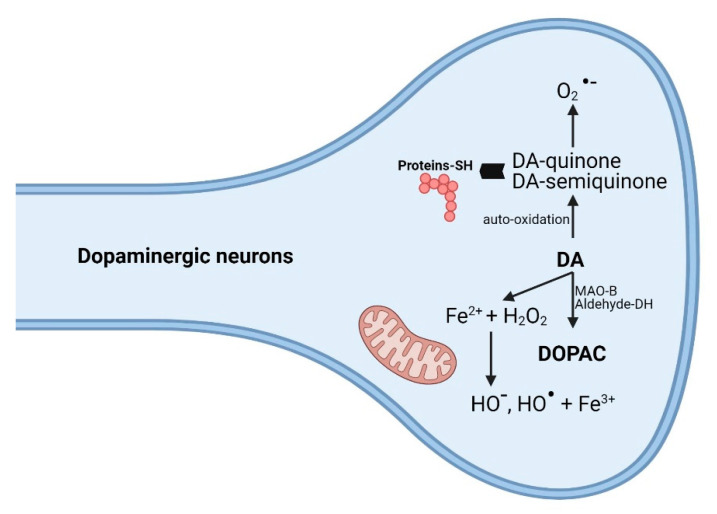
Toxic reactions of dopamine (DA) metabolites generated after oxidation. The neurotoxicity of DA is due to reactive oxygen species (ROS) produced as a result of monoamine oxidase (MAO) metabolism or as a result of an auto-oxidation process. 1. Under the action of MAO, 3,4-dihydroxyphenylacetic acid (DOPAC), and H_2_O_2_ are later transformed into ROS (OH^−^, OH^●^). within mitochondria. 2. The process of self-oxidation results in DA-quinones and DA-semiquinones which in turn generate ROS (O_2_^●−^). These metabolites also interact with the–SH groups of proteins with toxic cellular action by altering the structure and/or function of protein. Created with BioRender.com (accessed on 22 January 2021).

This interaction is beneficial for Siah1 as it is protected from degradation and can initiate the apoptotic process [134,151]. Other studies support the idea that tyrosine nitration is an important factor in the development of neurodegenerative diseases [149] including Parkinson’s disease [152]. Regarding free radicals, both ROS and RNS [153] are mediators of inflammation, but also the inflammatory process itself is a producer of ROS and RNS, thus increasing the susceptibility to neuronal degeneration, mediated by DA in the substantia nigra [154]. DA, uncontrollably released as a result of amphetamine abuse, is responsible for the excess of ROS. The main metabolization pathway of DA is via MAO-B, but another degradation pathway involves oxidation of the catechol nucleus, generating quinones and reactive species [155], as shown in Figure 6. These quinones can bind to cysteine thiol groups impairing normal protein functions [140].

### 3.8. Cocaine

The reason for cocaine induced neurotoxic effect is mainly derived from the mechanism of action, involving influencing neurotransmission by inhibiting mediator reuptake and inducing self-oxidation [134,156] to which hypoxia caused by vasoconstriction is added. The neurotoxic effect is amplified by the increased activity of the nuclear factor kappa B (NF-κB) [157] conducting to high levels of pro-inflammatory cytokines and oxidases. Studies in rodents show increased levels of DA in the NAc [158] following decreased reuptake mechanisms.

High levels of peroxidated lipids and increased antioxidant enzymatic activity (GPx, SOD) [150] in certain areas of the brain, *cortex* and *corpus striatum*, were observed [149,159]. Following chronic cocaine use, GPx activity in the *hippocampus* is abolished [137,160]. The neuronal membrane is impermeable to GSH, consequently, the intervention of astrocytes is compulsory. They possess a transport mechanism (xc^−^ cystine/glutamate antiporter) involving a non-vesicular release of GLU outside neuron cells in exchange for cystine, which is supposed to be sensitive to free radicals generated by abusive drug use [161]. Degradation products [162] of cocaine, norcocaine, and nitrogen derivatives (nitroxide, nitrosonium) [158] have a much higher oxidizing potential, supported by determinations made on rodent brain tissue, the affected areas being the *prefrontal cortex* (PFC), *corpus striatum*, and the *cerebellum* [158,163,164]. An elevation of the nitrite level in the PFC and in the NAc is also observed [165,166] as a consequence of increased NOS activity. It has been hypothesized that ROS are able to block DAT transporter leading to DA accumulation, which is oxidized and, consecutively, decreases the activity of antioxidant systems [167]. Moreover, the neuronal destruction occurring as a result of oxidative stress induced by cocaine abuse may increase the incidence of neurodegenerative diseases (Alzheimer’s, Parkinson’s) or psychiatric (schizophrenia, mania) ones [134,149,159]. An important argument to support this hypothesis comes from post-mortem determinations on the brain of cocaine abusers in which an increase in alpha-synuclein expression was observed as a result of DA neuron destruction [168]. The body synthesizes neurotrophins in order to protect neuronal tissue against the injuries caused by oxidative stress. Depending on the type of drug used, the expression Brain Derived Neurotrophic Factor (BDNF) differs, so that in methamphetamine and heroin abuse the BDNF level did not change compared with the control population. In MDMA and cocaine users, the concentration of neurotrophins increased but only for the initial period of abstinence, the values gradually returning to normal [169].

### 3.9. Antioxidant Therapy Related to Drug Abuse

This paragraph briefly discusses the antioxidant strategy for the drug-induced neurotoxicity presented in this review and possible pharmacological targets for treatment. It is expected that new, more effective and accessible therapies will be discussed in the near future [170].

The mechanism underlying the neurotoxicity induced by amphetamine derivatives is multifactorial, including the dysregulation of mitochondrial function, with a direct influence on neuronal energy balance and overproduction of ROS. In addition, the accumulation of DA in the synaptic cleft increases the sensitivity to oxidative stress and promotes the activation of apoptotic neuronal processes [170,171,172].

Thus, multiple in vivo and in vitro studies have been performed to discover compounds that reduce the production of ROS and RNS and also minimize neuronal degeneration. As follows, the antioxidant therapies used in these studies will be discussed.

One of the most commonly used antioxidants, ascorbic acid (vitamin C), has been shown to be beneficial in neurotoxicity that has arisen from the use of methamphetamine. In vitro, vitamin C reduced ROS levels and regulated the activity of molecules involved in the apoptotic process [173,174].

Other antioxidants used are tocopherols, especially alpha-tocopherol (vitamin E), which have the ability to protect the cell membrane against oxidative stress by stabilizing the phospholipidic bilayer against peroxidation. In addition, tocopherols have the ability to act against reactive species and thus reduce the level of MDA, generated as a result of lipid peroxidation [175,176].

Dietary selenium supplementation provides antioxidant protection, reduces markers of oxidative stress and restores the optimal GSH/GSSG ratio, results which are supported by in vitro studies [177,178]. However, although selenium is an essential microelement, it has a narrow therapeutic index, the excess being incriminated in the occurrence of toxicity; therefore, selenium supplementation requires caution [179,180,181]. An alternative to the regulation of mitochondrial function is the administration of “mood stabilizers”, such as lithium and valproic acid, as they are involved in the correction of the malfunction of the mitochondrial membrane, prevent the release cytochrome c and reduce the expression of proteins involved in the apoptotic process [182,183]. Further research to find substances that show the same tendency to decrease reactive species of nitrogen (RNS) [184] was conducted using neuronal nitric oxide synthase (nNOS) inhibitors, such as 7-nitroindazole (7-NI) [185,186], S-methylthiocitrulline or 3-bromo-7-nitroindazole [187]. The mechanism of action has not been fully elucidated and it is believed that neuroprotection is due to the ability to inhibit NO formation rather than involvement in thermoregulation, as methamphetamine induces hyperthermia [187].

Concerning the excitotoxicity induced by GLU by the facilitation of the intracellular influx of Ca^2+^, studies have been carried out using melatonin. This substance has protective properties against oxidative damage by regulating the intracellular level of Ca^2+^ [188,189].

N-acetylcysteine exerts its protective effect after the use of methamphetamine [190,191,192,193]; the proposed mechanism is the regulation of the level of GSH [194] and the control of the concentration of GLU; it is regulated following stimulation of the antiport cystine-glutamate [195]; regulation of the level of pro-inflammatory cytokines [196] also appears to be important. In addition, neuropeptide Y may have a protective role against neuronal apoptosis induced by methamphetamine via GLU [197].

New therapeutic approaches focus mainly on immunotherapy, aimed at reducing the amount of narcotic drugs that reach the CNS by synthesizing antibodies that bind to these illicit substances and limit their transfer and distribution [198,199]. However, there are some limitations given the fairly variable antibody titer [200] and the high costs [201].

The contribution of naturally occurring compounds to antioxidant therapy is a well-known trend in research. Thus, a series of molecules of natural origin have been identified and highlighted, which seem very promising both in effect and safety such as epigallocatechin gallate (EGCG) [202], sulforaphane [203], 1-methyl-1,2,3,4-tetrahydroisoquinoline (1MeTIQ) [204], resveratrol [205], 7,8-dehydroxyflavone (7,8-DHF) [206,207].

**Figure 7:**
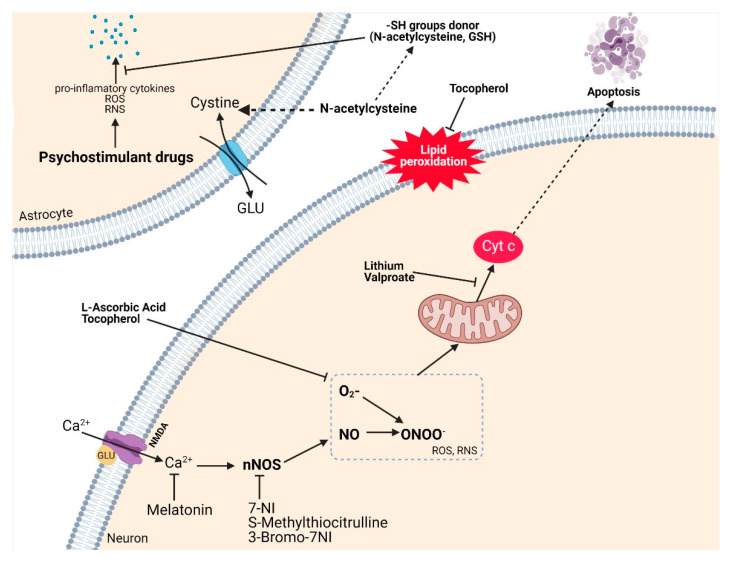
This illustration summarizes the proposed mechanism of antioxidant therapy in drug-related toxicity. Excessive stimulation of NMDA receptors by glutamate (GLU) generates numerous reactive oxygen (ROS) and/or nitrogen (RNS) species, subsequently leading to mitochondrial dysfunction, lipid peroxidation, and apoptosis. Thus, the use of compounds with antioxidant activity (e.g., L-ascorbic acid, tocopherol) reduces the oxidative status and preserves the integrity of the cell membrane. Lithium and valproic acid are involved in the correction of the malfunction of the mitochondrial membrane preventing the release of cytochrome c and reducing the expression of proteins involved in the apoptotic process. Neuronal nitric oxide synthase (nNOS) inhibitors limit the production of RNS. Melatonin protects against oxidative damage by regulating the intracellular level of Ca^2+^. N-acetylcysteine exerts its protective effect by regulating the level of GSH and controlling the concentration of GLU; following stimulation of the antiport cystine-glutamate and regulating the level of pro-inflammatory cytokines. Created with BioRender.com (accessed on 22 January 2021).

A limitation of the mechanisms proposed for the antioxidant substances used as scavengers for free radicals or inhibitors of the formation of ROS/RNS is that a large part of the experiments are based on in vitro studies (e.g., ascorbic acid, selenium, lithium, valproate); only a few results are based on in vivo experiments on animal models (e.g., N-acetylcysteine); this is why their relevance to clinical practice is uncertain (the evaluation of the effective dose in humans, transfer to the CNS, etc., still requires in-depth studies). The mentioned mechanisms proposed to explain the antioxidant action are highlighted in the Figure 7.

Antioxidant therapy is a promising approach to target neuronal damage and facilitate neuroprotection and, in the future, it could be included in all medical conditions caracterized by oxidative stress, supposedly even those caused by the abuse of recreational drugs.

## 4. Conclusions

Regardless of the abused drug, the brain is the most susceptible and vulnerable organ to neurotoxic action caused by oxidative stress mostly because of the high content of transition metals and lipids. It has been observed that psychoactive agents consumed produce not only structural but also behavioral changes. An interesting topic would be the question of what causes neurodegeneration over time. Certainly, one of the factors involved in the brain and whole-body aging is oxidative stress associated with poor folding and aggregation of proteins and dysfunction of calcium channels correlated with GLU-induced excitotoxicity. One important research topic is the mechanism of neurodegenerative diseases in the context of drug addiction as one hypothesis states that these conditions are directly correlated with oxidative stress induced neuroinflammation. The molecular mechanism of different neurological conditions is unknown, but the association between free radicals, aging, and neurodegeneration is confirmed. Another important aspect is drug addiction as it has been demonstrated that it increases the level of free radicals. All of these mechanisms have, at least at some stage, one element in common, namely DA. This neurotransmitter is involved in the process of generating ROS and RNS directly, as a result of oxidation processes, or indirectly by promoting GLU neurotransmission. The chemical structure of psychoactive compounds explains the potency but also the tropism towards certain areas of the CNS. However, their ability to influence DA neurotransmission is different. Although the mechanisms of neurotoxicity produced by psychoactive substances are extremely complex and are not yet fully understood, these toxic effects could, in theory, be avoided with the aid of antioxidant therapy.

## Figures and Tables

**Table 1:** In vitro dopamine and serotonin uptake transporter inhibition (IC_50_ values) and release data (EC_50_ values).

Compound	Monoamine Uptake Transporter Inhibition (IC_50_)				Monoamine Release (EC_50_)			
	DAT	SERT	DAT/SERT Ratio	Ref.	DAT	SERT	DAT/SERT Ratio	Ref.
Cathinone	14.0 (10–20) *	>100 *	>10 *	[9]	5.64 (3.0–10) *	>100 *	na	[9]
Methcathinone	1.12 (0.83–1.5) *	>10 *	>10 *	[9]	2.36 (1.7–3.3) *	>33 *	na	[9]
	2.4 (1.7–3.4) *	46 (30–71) *	19 (8.8–42) *	[64]	12.5 **	3.860 **	309 **	[56]
Mephedrone	3.31 (2.6–4.2) *	4.64 (3.7–5.9) *	1.4 (0.9–2.4) *	[9]	3.75 (1.7–8.4) *	5.98 (3.2–11) *	na	[9]
	1.4 (1.2–1.4) *	83 (66–104)	na	[65]	49.1 ± 8.32 **	118.3 ± 25.9 **	2.41 **	[10]
	5.7 (4.5–7.2) *	3.6 (2.8–4.6) *	0.63 (0.39–1.02) *	[66]				
	970 ± 50 **	310 ± 80 **	na	[7]				
	762 ± 79 **	422 ± 26 **	na	[10]	51 ± 5 **	122 ± 10 **	na	[10]
Buphedrone	4.24 (3.3–5.5) *	70 (2–2700) *	>10 *	[67]				
Pentedrone	2.5 (2.0–3.2) *	135 (5–3700) *	>10 *	[67]	>100 *	>100 *	na	[55]
	0.4 (0.3–0.4) *	16 (14–18) *	na	[65]				
Methedrone	35 (15–79) *	4.73(3.2–6.9) *	0.14 (0.04–0.46) *	[67]	506 **	120 **	0.24 **	[56]
Flephedrone	6.35 (4.2–9.5) *	>10 *	5.8 (0.8–41) *	[9]	12.5 (5.7–28) *	>33 *	na	[9]
					83.4 **	1290 **	15.4 **	[56]
3–FMC	1.7 (1.0–3.0) *	56 (7–472) *	>10 *	[67]				
Pyrovalone	0.035 (0.03–0.04) *	13.0 (10.8–15.8) *	>100 *	[9]				
α-PVP	0.2 (0.1–0.3) *	237 (196–291) *	na	[65]	>100 *	>100 *	na	[55]
	0.04 (0.01–0.1) *	>100 *	>1000 *	[64]				
	12.8 **	>10,000 **	>781**	[56]				
α-PPP	0.540 ± 0.076 *	188 ± 12 *	na	[55]	>10	>10	na	[55]
	196 **	>10,000 **	>51 **	[56]				
α-PVT	0.342 ± 0.0049 *	242 ± 41 *	na	[55]				
3,4-DMMC	9.4 (7.6–11.7) *	1.1 (0.9–1.4) *	0.12 (0.08–0.18) *	[66]				
Methylone	4.82 (3.8–6.1) *	15.5 (10–26) *	3.3 (1.5–6.8) *	[9]	>100 *	>10 *	na	[9]
	2.0 (1.7–2.3) *	68 (58–80) *	na	[65]	133.0 ± 11.2 **	242.1 ± 48.3 **	1.82 **	[8]
	560 ± 50 **	230 ± 30 **	na	[11]				
	12320 ± 133 **	1017 ± 59 **	na	[10]	117 ± 12 **	234 ± 35 **	na	[10]
Ethylone	5.68 (4.9–6.5) *	4.46 (3.8–5.2) *	0.8 (0.6–1.1) *	[9]	>100 *	9.9 (2.4–40) *	na	[9]
					>10 *	1.48 ± 0.25 *	na	[55]
Butylone	2.9 (2.5–3.4) *	6.22 (4.3–9.0) *	2.1 (1.3–3.6) *	[9]	>100 *	5.5 (1.8–17) *	na	[9]
	1710 ± 320 **	680 ± 130 **	na	[11]				
	400 ± 20 **	1430 ± 16 **	na	[68]	(−) **	330 ± 40 **	na	[68]
Pentylone	1.34 (1.0–1.7) *	8.37 (5.4–13) *	6.2 (3.2–13) *	[67]	>100 *	>100 *	na	[55]
	120 ± 10 **	1360 ± 100 **	na	[68]	(−) **	1030 ± 180 **	na	[68]
MDPBP	0.11 (0.07–0.16) *	15 (5.4–39) *	132 (34–557) *	[64]	>10 *	>10 *	na	[55]
MDPP	1.08 ± 0.1 *	126 ± 36 *	na	[69]	>10 *	>10 *	na	[55]
	0.53 (0.27–1.1) *	75 (49–114) *	141 (45–422) *	[64]				
MDPV	0.031 (0.03–0.04) *	9.3 (6.8–12.8) *	>100 *	[9]	>100 *	>100 *	na	[9]
	0.07 (0.07–0.08) *	4.5 (4.0–5.2) *	na	[65]				
	0.05 (0.04–0.06) *	9.6 (3.4–27) *	192 (57–675) *	[64]				
	4.1 ± 0.5 **	3305 ± 305 **	na	[10]	2.3 ± 0.8 **	(−) **	na	[10]

3-FMC = 3-Fluoromethcathinone; DAT = dopamine transporter; SERT = serotonin transporter; (−) = inactive; na = not reported by authors; DAT/SERT ratio = (DAT IC_50_)^−1^/(SERT IC_50_)^−1^, DAT/SERT ratio = (DAT EC_50_)^−1^/(SERT EC_50_)^−1^; values are expressed as mean or means ± SD, similar to original articles. The ability of tested drugs to inhibit the transport-mediated uptake is expressed as IC_50_. The ability of tested drugs to influence the transporter-mediated release is expressed as EC_50_. * in vitro studies for neurotransmitter reuptake inhibition and release using HEK293 (Human Embryonic Kidney 293) cell line expressing DAT and SERT (IC_50_ and EC_50_ values are expressed in μM). ** in vitro studies for neurotransmitter reuptake inhibition and release from rat brain synaptosomes (IC_50_ and EC_50_ values are expressed in nM).

**Table 2:** In vitro dopamine and serotonin uptake transporter inhibition (IC_50_ values) and release data (EC_50_ values).

Compound	Monoamine Uptake Transporter Inhibition (IC_50_)				Monoamine Release (EC_50_)			
	DAT	SERT	DAT/SERT Ratio	Ref.	DAT	SERT	DAT/SERT Ratio	Ref.
Amphetamine	1.3 (0.83–2.0) *	>10 *	>10 *	[9]	2.36 (1.7–3.3) *	>33 *	na	[9]
	1.3 (0.8–2.0) *	45 (24–85) *	35 (12–106) *	[55]				
	93 ± 17 **	3418 ± 314 **	na	[10]	5.8 ± 0.4 **	698 ± 71 **	na	[10]
Methamphetamine	1.05 (0.75–1.5) *	>10 *	>10 *	[9,67]	1.56 (0.9–2.8) *	>33 *	na	[9]
	1.1 (0.7–1.7) *	18 (3–116) *	17 (1.8–166) *	[64]	0.435 ± 0.075 *	23.3 ± 4.2 *	na	[55]
					8.5 ± 1.4 **	1291 ± 241.6 **	152.0 **	[8]
Ephedrine	46 (27–79) *	230 (72–735) *	5.0 (0.9–27) *	[64]				
MDMA	17 (12–24) *	1.36 (1.0–2.0) *	0.08 (0.04–0.16) *	[9]	22 (8.9–53) *	5.63 (3.5–9.2) *	na	[9]
	31(8–118) *	2.0 (1.4–3.0) *	0.06 (0.01–0.4) *	[64]	7.5 ± 2.3 *	1.1 ± 0.29 *	na	[55]
					51.2 ± 6.3 **	49.6 ± 5.4 **	0.97 **	[8]
MBDB	22 (20–26) *	2.04 (1.3–3.0) *	0.09 (0.05–0.15) *	[9]	>100 *	2.49 (1.0–6.9) *	na	[9]
MDEA	9.3 (8.0–11) *	1.27 (0.93–1.7) *	0.14 (0.01–0.21) *	[9]	>100 *	2.88 (1.6–5.0) *	na	[9]
Cocaine	0.768 (0.6–1.0) *	2.37 (2.0–2.9) *	3.1 (2.0–4.8) *	[9]	>100 *	>100 *	na	[9]

DAT = dopamine transporter; SERT = serotonin transporter; na = not reported by authors; DAT/SERT ratio = (DAT IC_50_)^−1^/(SERT IC_50_)^−1^, DAT/SERT ratio = (DAT EC_50_)^−1^/(SERT EC_50_)^−1^; values are expressed as mean or means ± SD, similar to original articles. The ability of tested drugs to inhibit the transport-mediated uptake is expressed as IC_50_. The ability of tested drugs to influence the transporter-mediated release is expressed as EC_50_. * in vitro studies for neurotransmitter reuptake inhibition and release using HEK293 (Human Embryonic Kidney 293) cell line expressing DAT and SERT (IC_50_ and EC_50_ values are expressed in μM). ** in vitro studies for neurotransmitter reuptake inhibition and release from rat brain synaptosomes (IC_50_ and EC_50_ values are expressed in nM).
